# The Roles of Dopamine D2 Receptor in the Social Hierarchy of Rodents and Primates

**DOI:** 10.1038/srep43348

**Published:** 2017-02-24

**Authors:** Yoshie Yamaguchi, Young-A. Lee, Akemi Kato, Emanuel Jas, Yukiori Goto

**Affiliations:** 1 Kyoto University, Primate Research Institute, Inuyama, Aichi, 484-8506, Japan; 2 Catholic University of Daegu, Department of Food Science & Nutrition, Gyeongsan, Gyeonbuk, 34830, South Korea; 3 Utrecht University, Graduate School of Natural Sciences, Princetonplein 5, 3584 CC, Utrecht, Netherland

## Abstract

Dopamine (DA) plays significant roles in regulation of social behavior. In social groups of humans and other animals, social hierarchy exists, which is determined by several behavioral characteristics such as aggression and impulsivity as well as social affiliations. In this study, we investigated the effects of pharmacological blockade of DA D2 receptor on social hierarchy of Japanese macaque and mouse social groups. We found acute administration of the D2 antagonist, sulpiride, in socially housed Japanese macaques attenuated social dominance when the drug was given to high social class macaques. A similar attenuation of social dominance was observed in high social class mice with D2 antagonist administration. In contrast, D2 antagonist administration in low social class macaque resulted in more stable social hierarchy of the group, whereas such effect was not observed in mouse social group. These results suggest that D2 receptor signaling may play important roles in establishment and maintenance of social hierarchy in social groups of several species of animals.

Dopamine (DA) is a neurotransmitter that has been shown to play roles in various aspects of brain function, depending on where in the brain it works^1^. DA signaling is processed thorough two distinct classes of receptors, D1-like and D2-like receptors^2^. DA D1 and D2 receptors often appear to yield the opposite effects in terms of behavioral outcomes, which are conserved across different species including rodents and non-human primates^3^,^4^,^5^. Recent studies have also demonstrated involvements of the DA system in regulation of social behaviors^6^,^7^,^8^,^9^, which may be partly associated with rewarding properties of social activity^10^,^11^.

Humans and many other animals live in social groups. Thus, social activity of subjects is practiced under the attributes of social groups such as social hierarchy and friendships. In particular, social hierarchy has been suggested a critical factor that affects reproduction and health of subjects living in social groups^12^. Social rank in hierarchy has been shown to be associated with several behavioral characteristics such as aggression and impulsivity, which are positively correlated, and common in both animals and humans^13^,^14^,^15^,^16^,^17^, although in humans, social affiliation, in addition to aggression and impulsivity, has been reported to be another behavioral strategy in gaining higher social rank in hierarchy^16^,^17^. In this regard, DA could play substantial roles in construction and maintenance of social hierarchy, as DA signaling has been demonstrated to be involved in regulation of aggressive and impulsive behavior^18^,^19^,^20^,^21^,^22^,^23^,^24^,^25^.

In this study, we examined the effects of pharmacological manipulation to inhibit D2 receptor function in socially housed macaques and mice. We specifically selected macaques and mice, since these species exhibit rigorous linear social hierarchy in their social groups, but distinct mechanisms seem to be involved in construction of social hierarchy between them. Associations between striatal D2 receptor expression and social rank or social status in non-human primates^26^ and humans^27^ have been already reported, with higher social rank/status subjects exhibiting higher striatal D2 receptor expression. Thus, we hypothesized that administration of the D2 receptor antagonist attenuated social dominance of drug-treated rodents and primates living in social groups.

## Results

### Individual behaviors of macaques housed in a social group

**Figure 1:**
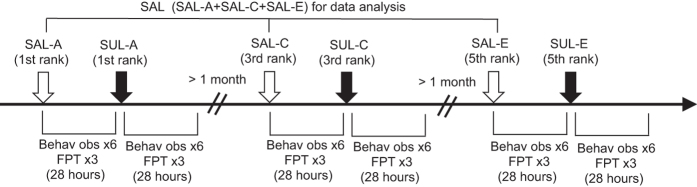
A design of the macaque experiments. A diagram illustrating timeline of experiment with drug administration in macaques. SAL: saline, SUL: sulpiride, FPT: food priority test, Behav obs: behavioral observation.

The effects of drug administration were examined in a social group consisting of 5 Japanese macaques. First, administration of the equivalent volume of saline to the 1st (SAL-A), 3rd (SAL-C), and 5th (SAL-E) rank subjects was examined as a control condition. Then, acute administration of the DA D2 receptor antagonist, (±)-sulpiride (SUL; 4.5 mg/kg), was given to the 1st (SUL-A), 3rd (SUL-C), and 5th (SUL-E) rank subjects sequentially (Fig. 1). Since no statistically significant difference with Friedman ANOVA was found in any of subjects across SAL-A, C, E administration conditions, data analysis was conducted with the SAL-A, C, E conditions combined into one group (n = 18 behavioral observations, with n = 6 times of behavioral observations in each of the SAL-A, C, E conditions; Fig. 1).

**Figure 2:**
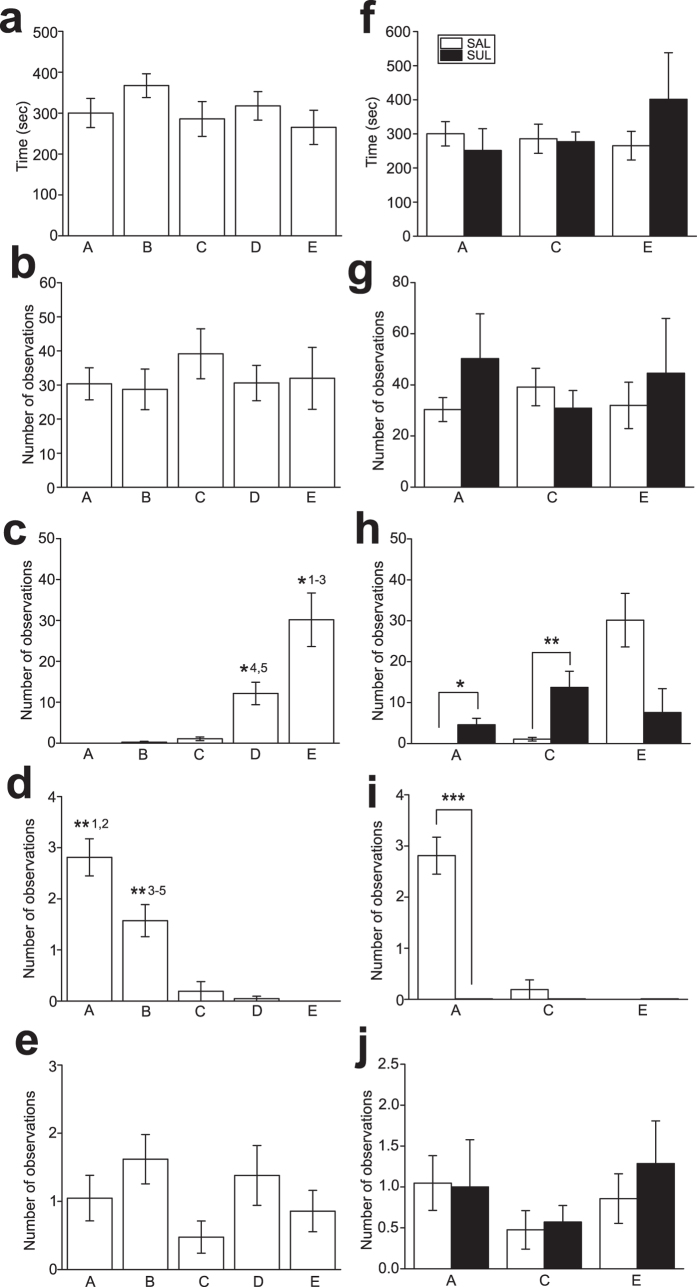
The effects of the D2 antagonist on individual behaviors of macaques. **(a–e)** Graphs showing locomotion (**a**), goal-directed actions (**b**), stereotypy (**c**), agonistic display (**d**), and scanning (**e**) of each macaque A to E in the group with SAL administration. Social rank is the highest in Subject A and getting lower to Subject E. Error bars indicate s.e.m. *^1–3^p < 0.001 vs. A–C, *^4^p = 0.014 vs. A, *^5^p = 0.029 vs. B, **^1^p = 0.018 vs. C, **^2^p = 0.036 vs. D, **^3–5^p = 0.018 vs. C–E. **(f–j)** Graphs showing locomotion (**f**), goal-directed actions (**g**), stereotypy (**h**), agonistic display (**i**), and scanning (**j**) of Subject A, B, and C with SUL administration. *p = 0.028, **p = 0.046, ***p = 0.043.

First, we assessed whether any individual behaviors were different in relation to social rank of subjects, with Kruskal-Wallis ANOVA. A duration of locomotion (Fig. 2a) as well as numbers of observations for goal-directed actions (Fig. 2b) and scanning (Fig. 2e) were not significantly different among subjects in the group. SUL administration did not alter these behavioral measurements in the drug-administered subjects, i.e., 1st, 3rd, and 5th rank subjects (Fig. 2f,g,j). In contrast, numbers of observations for stereotypy and agonistic display were found different depending on social rank of subjects. Thus, stereotypy was significantly or marginally significantly higher in lower rank than higher rank subjects (χ^2^ = 47.9, p < 0.001; p < 0.001 in 5th rank vs. 1st-3rd rank, p = 0.014 in 4th vs. 1st rank, p = 0.029 in 4th vs. 2nd rank; Fig. 2c). In contrast, agonist display was significantly or marginally significantly higher in higher rank than lower rank subjects (χ^2^ = 55.0, p < 0.001; p = 0.018 in 1st vs. 3rd rank, p = 0.036 in 1st vs. 4th rank, p = 0.053 in 1st vs. 5th rank, p =  0.018 in 2nd vs. 3–5th rank; Fig. 2d).

**Figure 3:**
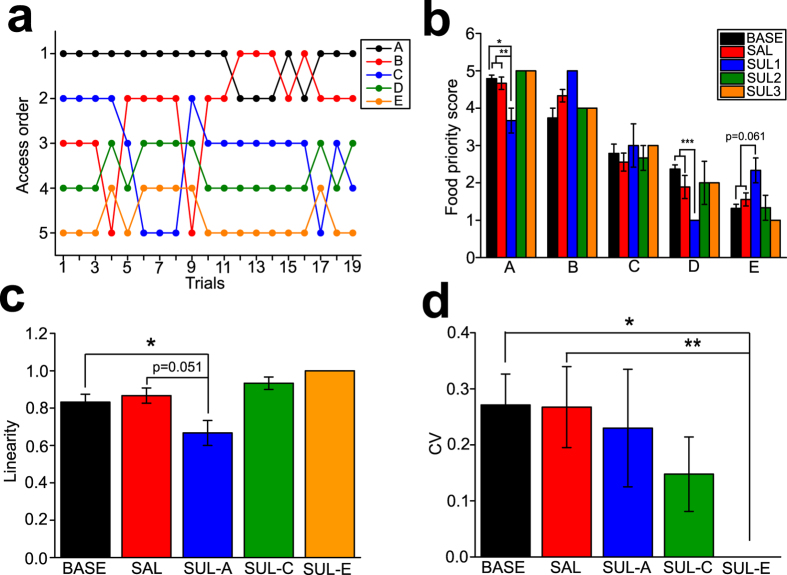
The effects of the D2 antagonist on social hierarchy of the macaque social group. **(a)** A graph showing orders of food accesses of each subject in the FPT at the BASE condition. **(b)** A graph showing FPT scores in the BASE condition and those with SAL and SUL administration to the 1st (SUL-A), 3rd (SUL-C), and 5th (SUL-E) rank subjects. Note that there were no variance on the FPS of some subjects, resulting in no error bars. *p = 0.006, **p = 0.050, ***p = 0.050. **(c)** A graph showing a linearity of social hierarchy expressed as coefficient of determination of linear regression. *p < 0.001. **(d)** A graph showing a stability of social rank of subjects expressed as coefficient of variation (CV) of FPT scores. *p = 0.043, **p = 0.043.

Then, the effects of SUL administration on individual behaviors in each of the drug-administered subjects were examined with Wilcoxon matched pairs test. Interestingly, significant changes of behaviors with SUL administration were observed only in the domains of individual behaviors that were dependent on social rank, i.e., stereotypy and agonist display. Thus, SUL administration significantly increased a number of stereotypy compared to that with SAL administration in the 1st (Z = 2.20, p = 0.028; Fig. 2h) and 3rd (Z = 1.99, p = 0.046; Fig. 2h) rank subjects, whereas SUL administration decreased, although it did not reach statistical significance, a number of stereotypy in the 5th rank subject (Z = 1.78, p = 0.075; Fig. 2h). SUL administration also significantly decreased a number of agonistic display in the 1st rank subject (Z = 2.02, p = 0.043; Fig. 2i).

These results suggest that some individual behaviors are social rank dependent, and these social rank dependent behaviors are associated with D2 receptor function.

### Social hierarchy of the macaque social group

Social rank of each macaque in the group was determined by the food priority test (FPT), similar to that described in other studies^28^. First, 19 trials at the frequency of one trial per day were conducted in the baseline (BASE) condition. In this group, social hierarchy appeared to be relatively unstable, as orders of access to foods by the subjects were variable in the BASE condition (Fig. 3a). However, in overall, a linear social hierarchy (expressed as Spearman R in each trial) was observed in the BASE condition (Fig. 3b,c).

Then, the effects of SAL and SUL administration for the 1st, 3rd, and 5th rank subjects were examined. Since no statistically significant difference was found for each of the SAL-A, C, E administration conditions, data analysis was conducted with the SAL-A, C, E conditions combined into one group (n = 9 times of the FPT, with n = 3 times of the FPT in each of the SAL-A, C, E conditions; Fig. 1). No difference of FPS scores in any subject between the BASE and SAL conditions was observed (Fig. 3b). A linearity of social hierarchy with SAL administration was also not different from that in the BASE condition (Fig. 3c).

SUL administration to the 1st rank subject resulted in a significant decrease of FPT scores in this drug-administered subject compared to the BASE conditions (Friedman ANOVA, χ^2^ = 6.00, p = 0.050; Wilcoxon matched pairs test, Z = 2.76, p = 0.006 vs. BASE; Z = 1.60, p = 0.11 vs. SAL; Fig. 3b). This treatment also caused FPT score alterations in non-drug administered cage mates at the same time, with significant difference of the scores among the BASE, SAL, and SUL conditions in the 4th rank subject (χ^2^ = 6.00, p = 0.050; Fig. 3b) and marginally significant difference in the 5th rank subject (χ^2^ = 5.60, p = 0.061; Fig. 3b). Linearity of social hierarchy expressed as Spearman R was significantly or marginally significantly lowered with SUL administration to the 1st rank subject compared to those in the BASE and SAL conditions (χ^2^ = 17.6, p < 0.001; Z = 3.58, p < 0.001 vs. BASE; Z = 1.95, p = 0.051 vs. SAL; Fig. 3c). SUL administration to the 3rd and 5th rank subject did not alter FPS scores (Fig. 3b) and linearity of social hierarchy (Fig. 3c) in the drug-administered subjects as well as non-drug administered cage mates. However, when SUL was administered to the 5th rank subject, a social hierarchy was more stabilized than other conditions, as variability of FPS scores expressed as coefficient of variation (CV) in all subjects in the group under the SUL5 condition was significantly lower than the BASE and SAL conditions (χ^2^ = 8.38, p = 0.079; Z = 2.02, p = 0.043 vs. BASE, SAL; Fig. 3e).

These results suggest that D2 receptor function is involved in social dominance of higher, but not lower, social rank subjects.

### Aggression in the macaque social group

**Figure 4:**
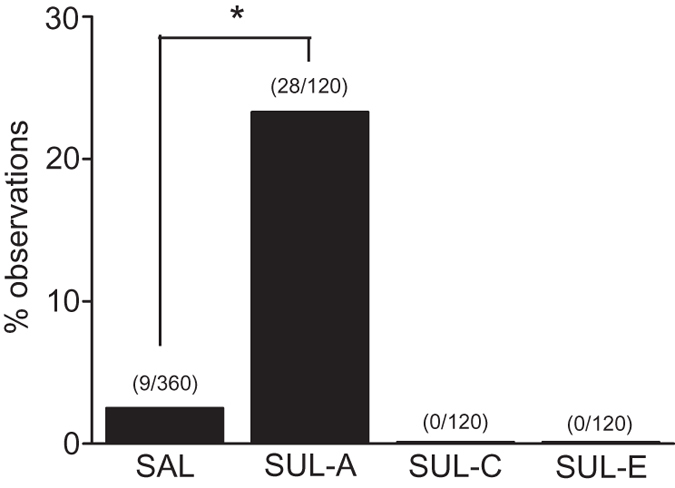
The effects of the D2 antagonist on aggression in the macaque social group. A graph shows percentage of observations in which aggression was noticed between any subjects in the social group with SAL and SUL administration at the 1st (SUL-A), 3rd (SUL-C), and 5th (SUL-E) rank subject. *p < 0.001.

Although aggression is also an important social behavior to determine social relationships among subjects in a social group, a number of observations for aggression in this group was very low throughout the experimental period, and therefore this behavioral observation was unable to be subjected for detailed analysis. However, in the SAL condition, a total of 360 observations for all subjects combined were conducted, of which aggression was present 9 observations. Under the condition of SUL administration to the 1st rank subject, aggression was present in 28 out of 120 observations, which was significantly higher number of observations than that in the SAL condition (Yates corrected χ^2^ = 52.0, p < 0.001; Fig. 4). On the other hand, no aggression was spotted in 120 observations each of SUL administration to the 3rd and 5th rank subjects, which was not significantly different from the SAL condition (Fig. 4).

These results suggest that lowering of social dominance by the 1st rank subject with SUL administration promotes stronger competitions among subjects in the social group.

### Social hierarchy of rodent social groups

A question has still remained whether and in what extent the effects of SUL on social hierarchy could be generalized and similar across different species. To address this issue, we have investigated the effects of SUL administration in socially housed mice, with each social group having 4 mice.

**Figure 5:**
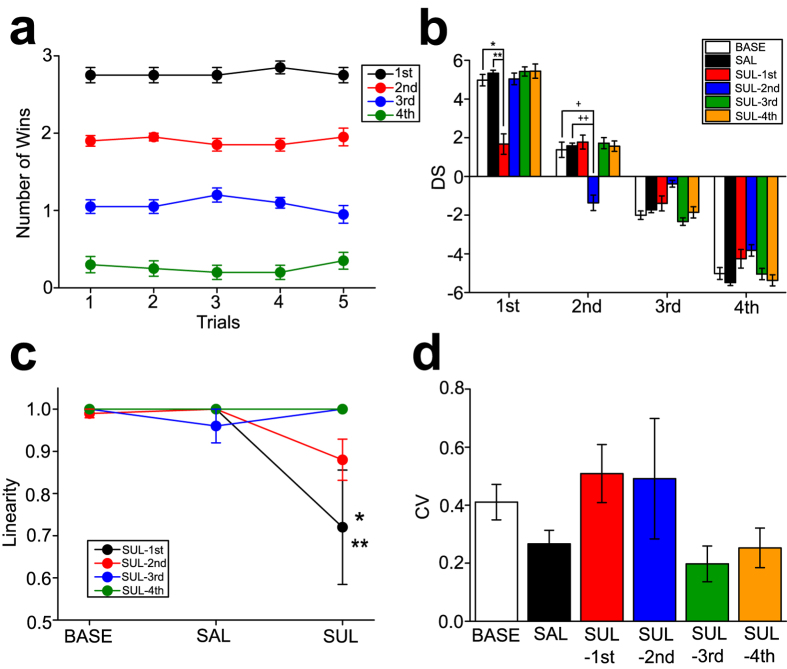
The effects of the D2 antagonist on social hierarchy of the mouse social groups. **(a)** A graph showing a number of wins for mice in each group in the tube rank test, with which stable hierarchy in mice social groups is illustrated. **(b)** A graph showing DS with D2 antagonist administration at each rank of mice. SUL-1st to SUL-4th indicate a set of groups in which the 1st to 4th rank mice, respectively, received drug administration. *p < 0.001, **p < 0.001, ^†^p < 0.001, ^††^p < 0.001. **(c)** A graph showing a linearity of social hierarchy expressed as Spearman R. *p < 0.003 vs. BASE in SUL-1st, **p = 0.003 vs. SAL in SUL-1st. **(d)** A graph showing a stability of social rank of subjects expressed as CV.

Social hierarchy of mice housed in groups was determined using the tube rank test, which was similar to that described in other studies^29^,^30^. Then, social dominance was quantified by David’s score (DS)^31^,^32^. In the mouse social groups, stable social hierarchy, unveiled by the tube rank test, was established in the no drug or saline treatment condition (i.e. BASE condition; Fig. 5a).

SUL (4.5 mg/kg, i.p.) or equivalent volume of SAL administration was given to the 1st-4th rank subjects in the groups, with a set of 5 groups for each rank. Twenty groups of 4 mice each (80 mice total), which were divided into 4 sets of drug administration conditions (thus n = 5 per condition), with each condition for drug administration in mice at different social rank, were examined. Three-way ANOVA with repeated measures, with Group (groups in which drug administration was given to either 1st, 2nd, 3rd, or 4th rank subjects) and Rank (1st vs. 2nd vs. 3rd vs. 4th) as between-subjects factors, and Treatment (BASE vs. SAL vs. SUL) as a within-subjects factor, have revealed no difference in Group (F_3,64_ = 0.313, p = 0.816), Treatment (F_2,128_ = 0.321, p = 0.726), and Group × Treatment interaction (F_6,128_ = 0.428, p = 0.859), but significant effects on Rank (F_3,64_ = 1070.8, p < 0.001), and Group ×  Rank (F_9,64_ = 2.209, p = 0.033), Treatment × Rank (F_6,128_ = 3.747, p = 0.002), and Treatment × Group × Rank (F_18,128_ = 5.565, p < 0.001) interaction. Post-hoc analysis have revealed that SUL administration to the 1st (p < 0.001 vs. BASE, SAL; Fig. 5b) and 2nd (p < 0.001 vs. BASE, SAL; Fig. 5b) rank subjects significantly reduced DS in these drug-administered subjects, without altering DS in other non-drug administered cage mates. A linearity of social hierarchy expressed as Spearman R was lowered when SUL administration was given to the 1st rank subject (two-way ANOVA with repeated measures; F_3,16_ = 2.69, p = 0.081 in Group; F_2,32_ = 6.33, p = 0.005 in Treatment; F_6,32_ = 3.47, p = 0.009 in Group x Treatment; p = 0.003 vs. BASE, SAL; Fig. 5c). Different from the macaque social group, SUL administration to lower social rank mice did not alter a stability expressed as CV of DS (Fig. 5d).

These results suggest that SUL administration attenuates social dominance in higher social rank, but not in lower social rank, mice, which is similar to the effects observed in macaques.

## Discussion

In this study, we found that D2 antagonist administration to higher social rank macaques and mice housed in social groups attenuated social dominance, whereas the same drug treatment did not alter social dominance in lower social rank animals. These findings are consistent with the studies showing that higher social class non-human primates^26^ and human subjects^27^ exhibit higher D2 receptor expression in the striatum. Thus, D2 receptor stimulation appears to promote some behavioral traits that are required to gain higher social class.

In contrast to dominant social class macaques, D2 antagonist administration had little influence on dominance of subordinate individuals. There are several possible explanations for this observation. For instance, since subordinate subjects were already low in dominance, ceiling effects may obscure social dominance-associated behavioral changes caused by the D2 antagonist. Alternately, since D2 receptor expression is lower in subordinates than that in dominant subjects^26^, even the same dose of drug administration may result in less effects in lower social status individuals than those at higher social status. The latter case may be particularly probable, since the dose of drug administration we utilized in this study was very low one.

Studies have demonstrated that higher social rank non-human primates are more aggressive and impulsive^13^,^14^,^15^,^16^,^17^, although in relation to impulsivity, this is not always true^12^ and could be even sometimes false^33^. Couppis and colleagues have reported that mice exhibiting higher D2 receptor expression are more aggressive than mice with lower D2 receptors^18^, which is consistent with the current findings. In contrast, D2 receptor stimulation and blockade by the agonist^24^ and antagonist^23^ have been shown to attenuate and increase impulsive behavior, respectively, which is opposite to what is expected from the suggested role of impulsivity in gaining higher social status. Thus, attenuation of social dominance by the D2 antagonist in this study may be explained by decreased aggression, but not impulsivity.

In this study, we compared macaque and mouse social groups, but the experimental conditions and methods to assess social hierarchy were substantially different. For instance, the compositions of the mouse (males) and macaque (males and females) groups, the experimental designs (longitudinal or repeated testing within the macaque group, cross-sectional testing of mouse groups), the separate tests for social hierarchy are quite different between species, and although macaques and mice are diurnal and nocturnal animals, respectively, experiments for both macaques and mice were conducted in the light cycle. Indeed, to compare these two species, it would be necessary to conduct the tests that are more comparable and several methods. This study also did not use state-of-the-art methods to assess dominance rank, linearity and stability^34^. In addition, due to the small sample size in the macaques, it is unclear the extent to which these findings are representative of populations of Japanese macaques. However, comparison of these species with utilization of different experimental conditions and procedures for each of mice and macaques would be still worthwhile. Thus, even different species of animals, social group conditions, and methods of testing social rank, the role of D2 function in social hierarchy construction was found very similar. In other words, the current finding demonstrates that such mutual roles of D2 function between mice and macaques is clearly independent of experimental conditions and methods. Moreover, our study suggests that the mutual role of D2 function between macaques and mice is still observed on top of whether the specific social group of Japanese macaques we used in the experiments is representative of Japanese macaques or not. Collectively, the finding on top of these various confounding factors emphasizes, but not discourages, the general roles of D2 function on social hierarchy. A future study with development of the methods based on ethological observations that can be applied commonly between rodents and macaques to determine their social hierarchy will be required to address these issues.

The effects of the D2 antagonist on social dominance were common in both rodents and non-human primates. However, the distinct effects of the D2 antagonist between rodents and non-human primates was still observed when the drug was given to the lowest rank subjects in the groups. Thus, when the D2 antagonist was treated in the lowest (5th) rank subject, social hierarchy was more stabilized without altering social rank of any subject in the group. Such observation was absent in rodent social groups. Stabilization of social hierarchy may bring some advantages to social groups such as social information transmission within groups^35^. The mechanisms of such stabilization have remained unclear; however, such effects may involve alterations of social affiliations between subjects in the group (Suppl. Results; Suppl. Fig. S1), which is consistent with the studies suggesting that social affiliation can be an important strategy to determine social hierarchy. Thus, such social affiliative strategy may play more substantial roles in non-human primate social groups, but this is less important in rodent social groups.

Some non-social behaviors such as stereotypy and agonistic display in non-human primates were found to depend on social rank, whereas other behaviors such as locomotion, goal-directed actions, and scanning were not. These social rank-dependent behaviors were modulated by the D2 antagonist, whereas the social-rank independent behaviors were not affected by the drug. Agonistic display was exhibited by high social rank, subjects. D2 antagonist administration attenuated agonistic display, suggesting that agonistic display may be associated with aggression. In contrast, stereotypy was observed in low social rank subjects. Moreover, modulation of stereotypy by the D2 antagonist was complex. In higher social rank subjects that did not exhibit stereotypy, D2 antagonist administration caused stereotypy, whereas D2 antagonist administration attenuated stereotypy in lower social rank subjects. Since stereotypy is promoted by increased DA release in the striatum^36^, one possible explanation for this observation would be that D2 antagonist administration lowers social dominance in higher social rank subjects, which would cause stress in these subjects. Such stress may in turn increase DA level^37^,^38^. In this regard, sulpiride has been shown to augment amphetamine-39,40 and cocaine-41 induced stereotypy, whereas this drug does not alter^42^,^43^ or even inhibits^44^ apomorphine-induced stereotypy. Thus, sulpiride may inhibit presynaptic D2 autoreceptor to augment DA release in higher social rank subjects that may experience stress, which may consequently promote stereotypy in higher social rank subjects. In lower social rank subjects that have relatively high DA level, sulpiride may mostly compete with DA binding on postsynaptic D2 receptors. This may also be somewhat consistent with the finding of biphasic effects of sulpiride, with low and high doses of sulpiride causing opposite behavioral changes^41^.

In conclusion, DA D2 receptor function may play an important role in animals living in hierarchical society, especially among high social class animals in groups, and this role is common across different species. In addition, in non-human primates, but not in rodents, lower D2 receptor function in low social class animals may also contribute for stabilization of social hierarchy.

## Methods

### Subjects

CD1 mice and Japanese macaques were used in this study. All experiments were conducted in accordance with the *Guidelines for Proper Conduct of Animal Experiments by the Science Council of Japan* and were approved by the Kyoto University Primate Research Institute animal ethics committee.

Adult male CD1 mice purchased from Charles-River Japan were housed in groups, with 4 mice per cage, until the end of experiments. These mice were different from those used in our previous study^45^, but housing and testing procedures in the current study were identical to those used in the previous study. These mice were 8 weeks old at the time of introduction, and experiments were conducted at 10–14 weeks. They had no kinship and were approximately equal weights and ages. Mice were housed in normal 12 hour light-dark cycle (the light cycle from 6:00 am to 6:00 pm), and experiments were conducted in the light cycle, although macaques are diurnal whereas mice are nocturnal animals.

A group of 5 (3 males, 2 females) Japanese macaques (*Macaca fuscata*) was used. These macaques were 4 years old, without kin relationship, and originated from geographically different areas. They had been housed in a cage as a group for approximately 2 years at the time of experiments. These macaques were housed in the cage outside of the building without no artificial lighting. The subjects were denoted as A-E based on the social rank in the BASE condition, from A for the highest to E for the lowest social rank. The weight and sex of each subject is summarized in the Suppl. Table S1. No relationship between social rank and weight or gender was observed.

### Behavioral observations of macaques

Behavioral observation was conducted with the focal animal sampling method. Two observers monitored and recorded behavior of each monkey for 15 minutes. Observation and recording were conducted 6 times (2, 4, 6, 24, 26, 28 hours) after saline (SAL) or the DA D2 receptor antagonist, sulpiride (SUL), administration (Fig. 1). Given that the study has shown that D2 receptor occupancy remains higher than 65% even 27 hours after SUL treatments in humans^46^, it was expected that each monkey who received SUL administration was still under the influence of the drug during 6 times of observations. We measured goal-directed action, stereotypy, agonistic display, scanning, locomotion, and aggression, of each animal in the group. Goal-directed action was defined as any behavior that an observer could predict a target to which a subject was approaching at least 1 sec before reaching the target, such as approaching to objects (e.g. water fountain), specific areas, or other mates in the cage. Stereotypy included repetitive circular running at the same orbit in the cage for more than twice or repeated licking of objects at constant speed for more than twice. Agonistic display was hard striking of any object in the cage or a part of the cage itself (e.g. wall, floor), which made loud noises more than twice repeatedly. Scanning was defined as sustained gazing at others for longer than 1 sec. Locomotion was calculated by subtraction of motionless time from the whole recording time. Aggression was biting, hitting, grabbing, or threatening with an open-mouth facial expression, by an attacker, which were simultaneously accompanied with bared-teeth display or scream by a recipient.

### Food priority test in macaques

The food priority test (FPT), similar to that described in other studies^28^, was conducted to assess social rank of each monkey in the group. The order of obtaining food (a portion of a sweet potato) given at roughly equal distance from all subjects was recorded in this test. After one subject obtained food, the next sweet potato portion was presented sequentially until the last subject obtained food. To emphasize the priority for accessing food, the size of the food was consecutively reduced to be approximately two-thirds smaller than the formerly presented food portion. First, 19 trials at the frequency of one trial per day were conducted in the baseline (BASE) condition. Then, the FPT was conducted 3 times at 2, 4, and 6 hours after each time of SAL or SUL administration (Fig. 1). Quantification of social rank was attempted by scoring (FPT score) for orders of food acquiring from 5 to 1 points with 5 points for the first access, 4 points for the second access, and so on to 1 point for the last access. Subjects in the group were denoted A-E, and social rank was determined by the FPS scores in the BASE condition, with the highest total score designated as 1st rank and “A”, and the lowest as 5th rank and “E”. Then, FPS scores in each trial were subjected for nonparametric regression analysis, and Spearman R was calculated for 19 trials in the BASE condition. Spearman R was used to express linearity of social hierarchy. Then, Spearman R was calculated for each trial in the SAL and SUL conditions, and the R values were compared between the conditions.

### Tube rank test in rodents

Social hierarchy of mice housed in groups was determined using the tube rank test, which was similar to that described in other studies^29^,^30^. The apparatus consisted of a transparent tube that was 30 cm long and 2.8 cm in diameter that was connected to transparent boxes at each end. There was a slit where a transparent wall was inserted to create a partition in the middle of the tube. After acclimation to the apparatus and training of mice to go across the tube from one end to the other end, pairs of mice from the same groups were simultaneously placed with one mouse into each of the boxes on either side of the tube. When mice from each side reached the middle of the tube, the partition wall was removed. The mouse that forced the other to retreat was designated as the “winner”, and the mouse that was retreated out of the tube was designated as the “loser”. A win and loss were scored by +1 and 0, respectively. Tournaments of all possible combinations of matches by pairs of mice in each group were conducted once per day at 5 times. Then, social dominance was quantified by David’s score (DS)^31^,^32^,^45^, with higher DS indicating more dominant. Briefly, DS was calculated by the following formula based on a dyadic proportion of wins and losses: *DS* = *w* + *w*_*2*_* − l − l*_*2*_, where *w* and *l* are the sum of the proportion of wins and losses of the subject, respectively, and *w*_*2*_ and *l*_*2*_ are the sum of the weighted proportion of wins and losses of the opponents that the subject wins/loses against, respectively.

### Drug administration

In the macaque experiments, acute SUL (4.5 mg/kg, dissolved in a few drops of 1N HCl and then diluted to 3.0 ml of 0.9% saline, i.m.) administration was given to the 1st, 3rd, and 5th rank subjects. The macaques that received drug administration was all males. SUL administration was given in one subject at each time, and sequentially from the 1st (SUL-A) to the 3rd (SUL-C), and then to the 5th (SUL-E) rank subjects (Fig. 1). SUL administration between each subject was separated at least one month. Administration of the equivalent volume of saline to the 1st (SAL-A), 3rd (SAL-C), and 5th (SAL-E) rank subjects was also examined as a control condition beforehand of SUL administration.

In the mouse experiments, SUL administration at the dose of 4.5 mg/kg in 0.3 ml of 0.9% saline was given intraperitoneally. First, SAL administration was given once per day for 5 days in their home cages, and then the tube rank test was conducted for 5 days. After confirming no change in social rank with SAL administration, SUL administration was given once per day for 5 days in the mouse that had received SAL administration beforehand. After 5 days of repeated SUL administration, the tube rank test was conducted again. A schematic diagram of the experimental design identical to that conducted in this study, except that D1, instead of D2, antagonist was administered, is shown in our recent another study^45^.

The dose of the drug was determined based on previous studies^47^,^48^,^49^, such that the dose of the drug could be the lowest to still yield clear behavioral effects, but did not cause sedation or motor control problems, both in rodents and non-human primates, since, if higher doses that cause side effects had been frequently observed, interpretation of the results would have been unclear whether the observations were really associated with functional deficits or consequence of the side effects. In fact, locomotion of mice for 20 minutes in the open field chamber with SUL administration at the dose of 4.5 mg/kg (14.1 ± 2.48 m; n = 16) was found not significantly different from that with SAL administration (13.7 ± 2.38 m; n = 15).

### Data analysis

Investigators who were not blinded to the experimental conditions conducted data collection and statistical analyses. Inter-rater reliability (Cohen’s kappa) between two blinded and two not blinded experimenters was calculated based on rating of 10 randomly selected video-recordings. Cohen’s kappas for aggressions and affiliations were approximately 0.80–0.95^45^, and those for agonistic display and stereotypy were 1.00 (confidence interval of 1.00–1.00) and 0.82 (confidence interval of 0.67–0.97), respectively. No data points were removed from statistical analysis without any specific reason. Sample sizes were not statistically predetermined. All statistical analyses were conducted using Statistica software. A probability value of p < 0.05 was considered as statistical significance.

## Additional Information

**How to cite this article**: Yamaguchi, Y. *et al*. The Roles of Dopamine D2 Receptor in the Social Hierarchy of Rodents and Primates. *Sci. Rep.* **7**, 43348; doi: 10.1038/srep43348 (2017).

**Publisher's note:** Springer Nature remains neutral with regard to jurisdictional claims in published maps and institutional affiliations.

## Supplementary Material

Supplementary Information
