# Supplementary material for: The Roles of Dopamine D2 Receptor in the Social Hierarchy of Rodents and Primates
Source: Sci Rep. 2017 Feb 24;7:43348. doi: 10.1038/srep43348 (PMC5324123; doi:10.1038/srep43348)
Supplement: Supplementary Information [file srep43348-s1.pdf]

Supplemental Information

The Roles of Dopamine D2 Receptor in the Social Hierarchy of Rodents and Primates

Y. Yamaguchi, Y-A. Lee, A. Kato, E. Jas, & Y. Goto

Supplemental Results

Supplemental Figure S1

Supplementary Table S1

Supplemental Results

Social affiliation in the macaque social group

We further investigated the effects of SUL administration on social affiliations, defined as either proximate sitting (sitting with the body touching to other cage mates, or less than a 30-cm distance between subjects, for longer than 1 sec), (2) grooming (for longer than 1 sec), or (3) playing (biting, hitting, grabbing, but without facial expressions associated with aggression), between subjects in the group. SUL administration to the 1st and 3rd rank subjects did not cause significant changes in social affiliations (Suppl. Fig. S1a-f). However, SUL administration to the 5th rank subject resulted in no change of social affiliations in the drug-administered subject, but facilitated affiliative contacts from the 3rd to 4th rank subjects (Wilcoxon matched pairs test, $Z=2.20$, $p=0.028$ vs. SAL; Suppl. Fig. S1d) and from the 4th to 3rd subjects ($Z=2.02$, $p=0.043$ vs. SAL; Suppl. Fig. S1e).

Considering the effects of SUL administration to the 5th rank subject that caused stabilization of social hierarchy, these results suggest that facilitation of the reciprocal affiliative connection between the 3rd and 4th rank subjects, along with further decreased aggression of this drug-administered subject, and reduced conflicts among the middle to low social class (3rd-5th rank) subjects, may be involved in such social hierarchy stabilization in this social group.

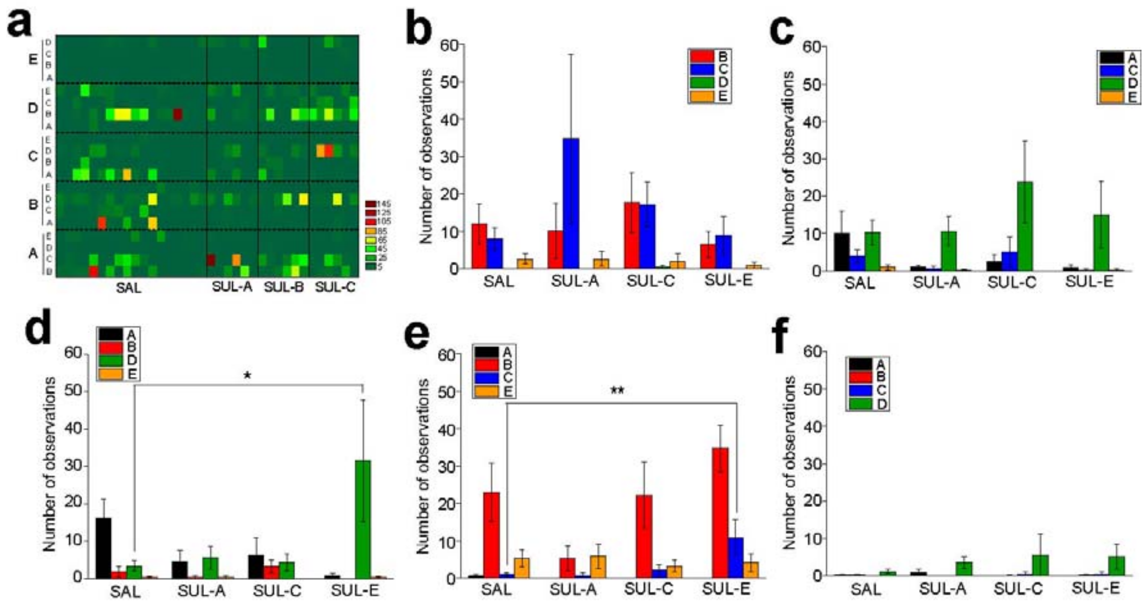

Supplementary Figure S1. The effects of the D2 antagonist on social affiliations in the macaque social group.

(a) A color-coded graph showing frequencies of social affiliations between subjects in each observation with SAL and SUL administration to the 1st (SUL-A), 3rd (SUL-C), and 5th (SUL-E) rank subjects. Initiators are shown larger font size on the left, and smaller font size next to them are receivers of affiliative contacts. **(b-f)** Graphs showing social affiliations separately for Subject A (b), B (c), C (d), D (e), and E (f). Receivers of affiliative contacts are shown with different colors. *p=0.028, **p=0.043.

Supplementary Table S1. Weights and sex of Japanese macaques used in this study.

Subject	Weights (kg)	Sex
A	6.2	♂
B	4.9	♀
C	7.9	♂
D	6.2	♀
E	5.1	♂
